# Endobronchial Tumor With Ball Valve Mechanism: A Real Airway Emergency

**DOI:** 10.7759/cureus.15522

**Published:** 2021-06-08

**Authors:** Andrew Talon, Muhammad Arif, Sreeja Biswas, Saad Alkhider, Ali Saeed

**Affiliations:** 1 Internal Medicine, Creighton University School of Medicine, St. Joseph's Hospital and Medical Center, Phoenix, USA; 2 Pulmonology and Critical Care, Mayo Clinic, Phoenix , USA; 3 Internal Medicine, Creighton University School of Medicine, St. Joseph's Hospital and Medical Center, Phoenix , USA; 4 Interventional Pulmonology, Norton Thoracic Institute, Phoenix , USA

**Keywords:** central airway obstruction, icast stent, tracheal tumor, interventional pulmonology, airway emergency, flexible bronchoscopy, pulmonary critical care, renal cell carcinoma (rcc), airway stent

## Abstract

Central airway obstruction due to the tumor can present as near-complete tracheal obstruction. The results can be life-threatening requiring emergent intervention. Rigid bronchoscopy has been preferred for the management of central airway obstruction. However, there are relatively few studies comparing rigid bronchoscopy and flexible bronchoscopy in treating these cases. We describe a 61-year-old woman with a lower trachea tumor with ball valve occlusion of the left mainstem bronchus and complete occlusion of the right mainstem bronchus successfully managed with flexible bronchoscopy and iCAST^® ^stent. We herein highlight the role of therapeutic flexible bronchoscopy with airway stenting as an efficacious treatment modality for the management of malignant central airway obstruction.

## Introduction

Central airway obstruction refers to obstruction involving the trachea or mainstem bronchi [1]. Malignant central airway obstruction occurs due to intraluminal tumor invasion or extrinsic tumor compression [1-2]. When these tumors present as near-complete tracheal obstruction, the results can be life-threatening, requiring emergent tumor debulking or airway stenting by trained specialists. There has been much debate on the upfront therapeutic use of flexible bronchoscopy over rigid bronchoscopy to restore airway patency in central airway obstructions. Rigid bronchoscopy has been always recommended as the procedure of choice in any patient where there is a doubt to secure the airway [2]. We present a case of malignant central airway obstruction with a ball valve mechanism involving the left main stem in a patient with renal cell carcinoma, which was successfully treated with flexible bronchoscopy and airway stenting. 

## Case presentation

**Figure 1:**
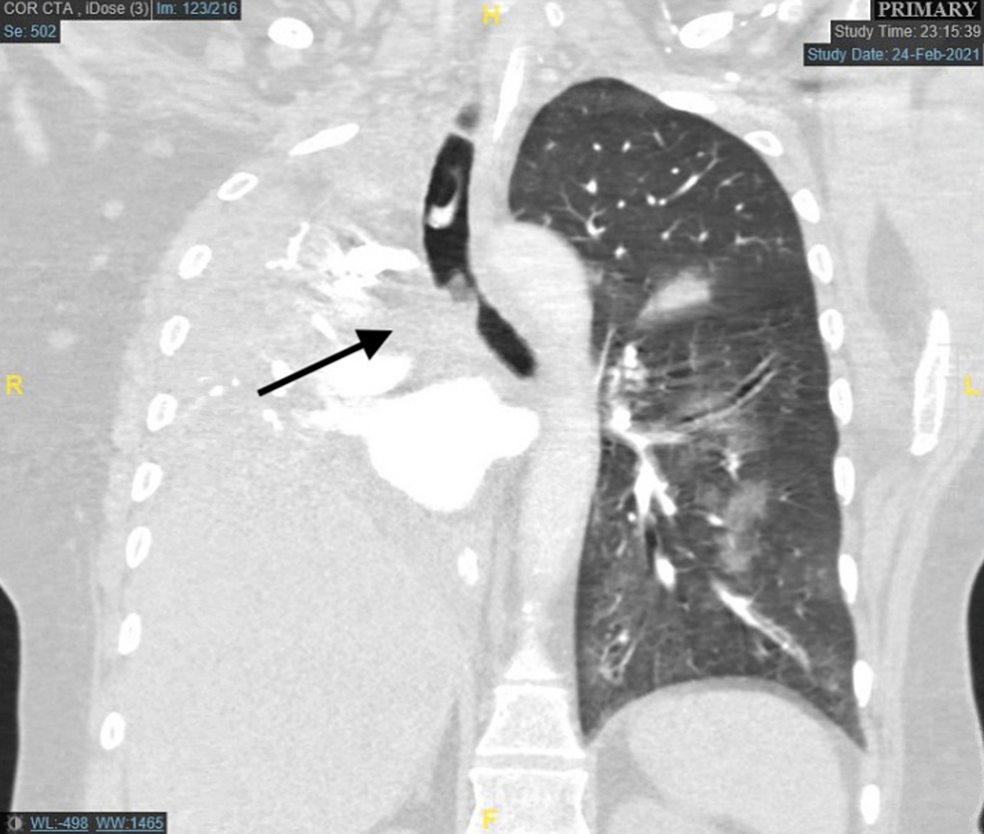
Chest computed tomography showing a right hilar mass invading the right main stem and lower trachea with complete right lung collapse.

A 61-year-old woman with chronic obstructive pulmonary disease (COPD) and a recent diagnosis of a right hilar mass presented with four weeks of progressively worsening dyspnea and hemoptysis. She underwent emergent intubation for impending respiratory arrest. Chest computed tomography (CT) confirmed a large right hilar mass invading the right main stem and lower trachea with complete right lung collapse (Figure 1). The patient was transferred to our facility two days later for interventional bronchoscopy to restore airway patency. On arrival, the patient was ventilated with pressure-regulated volume control (PRVC) mode of ventilation, tidal volume 6 mL/kg ideal body weight, a fraction of inspired oxygen (FiO2) 100%, rate 14 per minute, positive end-expiratory pressure (PEEP) of 8 cm H2O. Arterial blood gas showed pH 7.21, partial pressure of carbon dioxide (pCO2) 67 mmHg, and partial pressure of oxygen (pO2) 70 mmHg. She was also noted to have high minute ventilation.

On flexible bronchoscopy, a flailing tumor was seen in the lower trachea with ball valve occlusion of the left mainstem bronchus and complete occlusion of the right mainstem bronchus (Video 1). Endoscopic resection was accomplished using electrocautery snare and cryoprobe (Video 1). The tumor originated from the right upper lobe (RUL) with architectural loss. Furthermore, the distal bronchus intermedius (BI) showed evidence of extrinsic compression with patent right middle and lower lobe segmental airways. 

**Video 1:** Endotracheal tumor with ball valve occlusion of the left mainstem bronchus. Bronchoscopic resection was accomplished using electrocautery snare and cryoprobe. A fully covered 7 x 22 mm iCAST® stent was placed at the bronchus intermedius. Argon plasma coagulation (APC) and T-1470 LiteTouch convergent laser® were used for hemostasis and tumor destruction.

**Figure 2:**
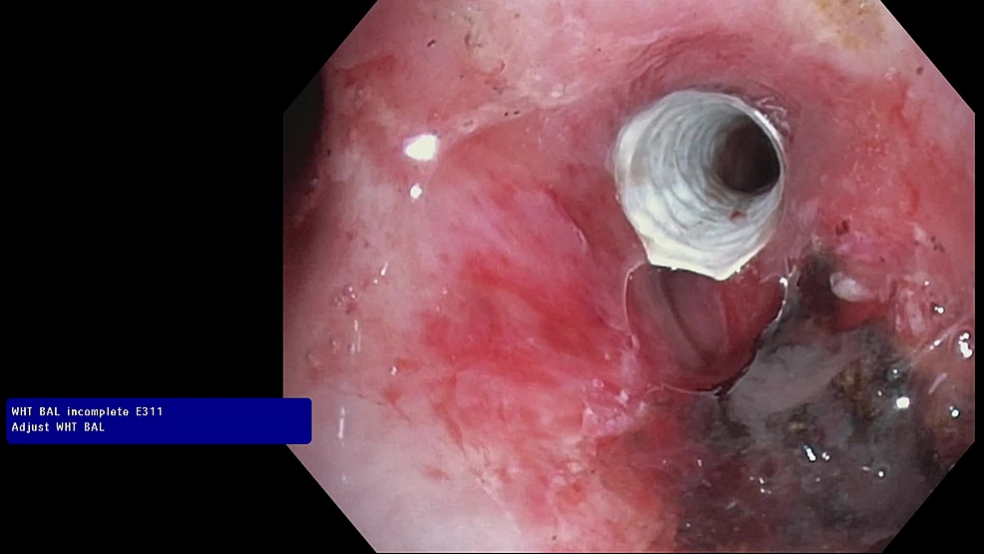
A 7 x 22 mm balloon-expandable fully covered stainless-steel iCAST® stent placed at the bronchus intermedius due to evidence of mixed obstruction (intraluminal tumor growth and extraluminal compression).

A 7 x 22 mm iCAST® stent was placed and upsized with a balloon to 9 mm at the BI (Figure 2). Argon plasma coagulation (APC) and T-1470 LiteTouch convergent laser® were used for hemostasis and tumor destruction at the RUL (Video 1). A biopsy of the tumor was taken which showed metastatic high grade sarcomatoid renal cell carcinoma. The patient was discharged on chemotherapy with concurrent palliative radiation therapy. She was later started on immunotherapy with Ipilimumab and Nivolumab. 

## Discussion

Our patient presented with a large tumor in the central airway resulting in respiratory failure. A tumor of this size may present as a medical emergency due to the risk of complete airway occlusion. Fortunately, the airway was secured by endotracheal intubation and airway patency was restored by flexible bronchoscopy.

Rigid bronchoscopy has historically been preferred to secure the airway in patients with central airway obstruction [1-3]. Rigid bronchoscopy allows excellent control of oxygenation and ventilation and provides a passageway for various therapeutic tools [1-3]. However, rigid bronchoscopy was not used in our case due to high minute ventilation compensating for mixed respiratory and metabolic acidosis. High minute ventilation would make mechanical coring with the rigid bronchoscope difficult to accomplish due to the risk of trauma to surrounding structures (eg, tracheal or bronchial tears). A retrospective study reviewed 775 cases of rigid bronchoscopy, and the results showed that lesions at or near the carina (such as our patient) have the highest correlation with procedural complications [4]. In this study, the authors discussed that since carinal lesions usually affect both bronchi, if these lesions bleed at the same time, it may be futile to use rigid bronchoscopy to tamponade the bleed, since ventilation would be impaired regardless in both lungs [4]. The patient did receive one ampule of bicarbonate to reduce minute ventilation and provide time for piecemeal removal of the flailing tumor in the lower trachea. Furthermore, the patient's underlying COPD and ball valve mainstem occlusion made her a high risk for hyperinflation and hypercarbia if rigid bronchoscopy were to be used. Removal of the tumor with cryoprobe was preferred due to the risk of asphyxia intraoperatively if forceps were used instead. Laser or APC could not be utilized upfront for tumor coagulation due to high oxygen requirements.

In certain situations, venovenous extracorporeal membrane oxygenation (VV ECMO) has been used to provide airway security for central airway obstruction [5]. VV-ECMO was not initiated on our patient since her airway was already secured by an endotracheal tube and oxygenation remained stable on PRVC mode of ventilation. VV ECMO would also carry a higher risk of life-threatening bleed, which remains one of the most fatal complications with ECMO [5].

This case is unique for several reasons. Firstly, the patient was at high risk of asphyxiation due to tumor occlusion of the left airway and bleeding associated with tumor excision. Tumor retrieval with flexible bronchoscopy may take longer, but there is no interruption in ventilation, which proved to be beneficial for our patient. Secondly, the use of flexible bronchoscopy initially allowed us to efficiently place a stent following debulking. Stenting was indicated in our case for mixed obstruction (extrinsic compression and intraluminal obstruction) with viable distal airways. In the current report, we want to highlight flexible bronchoscopy as an alternative in severely acidotic patients as it provides more efficient "flexible" options. 

## Conclusions

Flexible bronchoscopy can be safely utilized in the resection of large endotracheal tumors with a high risk of tumor dislodgement, and potential of ventilation failure, in patients with metabolic acidosis. Currently, randomized research comparing flexible bronchoscopy and rigid bronchoscopy in the treatment of malignant central airway obstruction is lacking. Randomized research may be possible in the future in a careful selection of stable patients with secured airways. 
